# Recent Applications of Artificial Intelligence from Histopathologic Image-Based Prediction of Microsatellite Instability in Solid Cancers: A Systematic Review

**DOI:** 10.3390/cancers14112590

**Published:** 2022-05-24

**Authors:** Mohammad Rizwan Alam, Jamshid Abdul-Ghafar, Kwangil Yim, Nishant Thakur, Sung Hak Lee, Hyun-Jong Jang, Chan Kwon Jung, Yosep Chong

**Affiliations:** 1 Department of Hospital Pathology, College of Medicine, The Catholic University of Korea, Seoul 06591, Korea; rizwan@catholic.ac.kr (M.R.A.); jamshid@catholic.ac.kr (J.A.-G.); kangse_manse@catholic.ac.kr (K.Y.); nishantbiotech2014@gmail.com (N.T.); hakjjang@catholic.ac.kr (S.H.L.); ckjung@catholic.ac.kr (C.K.J.); 2 Catholic Big Data Integration Center, Department of Physiology, College of Medicine, The Catholic University of Korea, Seoul 06591, Korea; hjjang@catholic.ac.kr

**Keywords:** artificial intelligence, neoplasm, microsatellite instability, deep learning, systematic review, whole slide images

## Abstract

**Simple Summary:**

Although the evaluation of microsatellite instability (MSI) is important for immunotherapy, it is not feasible to test MSI in all cancers due to the additional cost and time. Recently, artificial intelligence (AI)-based MSI prediction models from whole slide images (WSIs) are being developed and have shown promising results. However, these models are still at their elementary level, with limited data for validation. This study aimed to assess the current status of AI applications to WSI-based MSI prediction and to suggest a better study design. The performance of the MSI prediction models were promising, but a small dataset, lack of external validation, and lack of a multiethnic population dataset were the major limitations. Through a combination with high-sensitivity tests such as polymerase chain reaction and immunohistochemical stains, AI-based MSI prediction models with a high performance and appropriate large datasets will reduce the cost and time for MSI testing and will be able to enhance the immunotherapy treatment process in the near future.

**Abstract:**

Cancers with high microsatellite instability (MSI-H) have a better prognosis and respond well to immunotherapy. However, MSI is not tested in all cancers because of the additional costs and time of diagnosis. Therefore, artificial intelligence (AI)-based models have been recently developed to evaluate MSI from whole slide images (WSIs). Here, we aimed to assess the current state of AI application to predict MSI based on WSIs analysis in MSI-related cancers and suggest a better study design for future studies. Studies were searched in online databases and screened by reference type, and only the full texts of eligible studies were reviewed. The included 14 studies were published between 2018 and 2021, and most of the publications were from developed countries. The commonly used dataset is The Cancer Genome Atlas dataset. Colorectal cancer (CRC) was the most common type of cancer studied, followed by endometrial, gastric, and ovarian cancers. The AI models have shown the potential to predict MSI with the highest AUC of 0.93 in the case of CRC. The relatively limited scale of datasets and lack of external validation were the limitations of most studies. Future studies with larger datasets are required to implicate AI models in routine diagnostic practice for MSI prediction.

## 1. Introduction

Colorectal cancers (CRCs) with high microsatellite instability (MSI-H) have a better prognosis and respond very well to immunotherapy [1,2,3]. MSI-H cancers generally show certain distinctive clinicopathological features, such as younger age, tumor location in the ascending colon, histologic features of mucinous or areas of signet ring cells, and tumor-infiltrating lymphocytes [4,5]. Microsatellite instability (MSI) is induced by somatic inactivation of mismatch repair genes, and it is approximately 15% in CRC, including sporadic (12%) and germline mutations (Lynch syndrome, 3%) [6,7,8,9]. CRC carcinogenesis also follows the chromosomal instability pathway, which is accompanied by the loss of heterozygosity (LOH) and chromosomal rearrangement [10]. Circulating tumor DNA (ctDNA) may be detected as LOH in DNA microsatellites, and it is also useful in detecting molecular heterogeneity [11]. Moreover, MSI-H has been observed in many other solid cancers, such as endometrial, gastric, breast, prostate, and pancreatic cancers [2,12,13]. The European Society for Medical Oncology (ESMO) also recommended the testing of the BRCA1/2 gene mutation and MSI-H in patients with metastatic castration-resistant prostate cancer, as it is related to the predictivity of therapeutic success [14,15].

Recently, immunotherapy has emerged as a promising approach for the treatment of malignancy, with many tumor-infiltrating lymphocytes such as metastatic melanoma, lung cancer, and other MSI-H cancers [3,16,17,18]. As melanoma has high immunogenicity and an abundance of adjacent immune cells, immunotherapy has been shown to be effective [19,20]. Similarly to melanoma, MSI-H cancers show abundant infiltrating lymphocytes and can also be a target for immunotherapy [21,22]. Because of this broad clinical importance, testing for MSI or mismatch repair deficiency (dMMR) has been recommended for more cancer types [23,24]. Moreover, the guidelines of many scientific societies recommend testing the MSI/dMMR universally [25].

MSI is not tested unanimously in all cancers due to the additional cost and time for molecular tests such as polymerase chain reaction (PCR) or immunohistochemistry (IHC), and sometimes it may also require additional biopsy [26,27,28,29,30]. Moreover, the results of MSI/dMMR are not fully reliable, as previous studies reported various sensitivity ranges for IHC and PCR (85–100% and 67–100%, respectively) [31,32,33]. A recent review article reported the discordance rate between IHC and PCR to be as high as 1–10% [10]. MSI/dMMR identification using only one method might lead to misinterpretation and using both methods can raise the cost [34]. In addition, immunotherapy itself is also costly and shows beneficial effects only in the MSI-H cancers; therefore, the accurate identification of eligible patients is important [35]. Owing to these limitations, a more robust and universally applicable method is required to predict the MSI with high accuracy and low cost.

Recently, artificial intelligence (AI)-based models were developed to predict MSI from hematoxylin and eosin (H&E) whole-slide images (WSIs), and have shown promising results [29,30]. AI-based models are emerging in many medical fields, including radiology, dermatology, ophthalmology, and pathology, with promising results [36,37,38,39,40]. In pathology, deep learning- (DL) based models have also shown surprising results in cancer detection, classification, and grading [29,41,42,43,44]. More recently, AI models are now being applied, even to molecular subtyping and treatment response prediction that surpasses human ability and can change the whole pathology practice in the future [44,45]. Pathologists have tried to find out the characteristic morphological features of MSI-H cancers such as tumor-infiltrating lymphocytes and mucinous morphology on H&E stained slides. However, it is hard to quantify these features manually, and the interpretation can vary widely according to the observers. To overcome these limitations, researchers started to develop AI models that can predict MSI status using the WSIs from many cancers [29,46,47]. Currently, AI technology for MSI prediction is at the basic level and the training data is still insufficient for validation.

Therefore, we designed a systematic review to assess the current status of AI application on the MSI prediction using WSIs analysis and to suggest a better study design for future studies.

## 2. Materials and Methods

### 2.1. Search Strategy

The protocol of this systematic review follows the standard guidelines for a systematic review of the Preferred Reporting Items for Systematic Reviews and Meta-Analyses (PRISMA) statement. A systematic search of online databases including EMBASE, MEDLINE, and Cochrane was conducted. Articles published in English up to August 2021 were included. The following queries were used in the search; “deep learning”, “microsatellite instability”, “gene mutation”, “prognosis prediction”, ”solid cancers”, “whole slide image”, “image analysis”, “artificial intelligence”, and “machine learning”. We also manually searched the eligible studies, and the included studies were managed using EndNote (ver. 20.0.1, Bld. 15043, Thomson Reuters, New York, NY, USA). The protocol of this systematic review is registered with PROSPERO (282422).The Institutional Review Board of the Catholic University of Korea approved the ethical clearance for this study (UC21ZISI0129).

### 2.2. Article Selection and Data Extraction and Analysis

The combined search results from online databases were retrieved and transferred to the EndNote, and duplicates were removed. Original studies with full text on AI and MSI prediction from WSIs in solid cancers were included. To identify eligible studies, two independent reviewers (MRA and YC) first screened the studies by title and abstract. Finally, the full text of each eligible study was reviewed. Any discrepancy between the authors (MRA and YC) regarding study selection was resolved by consulting a third author (JAG). Case studies, editorials, conference proceedings, letters to the editor, review articles, poster presentations, and articles not written in English were excluded.

## 3. Results

### 3.1. Characteristics of Eligible Study

**Figure 1:**
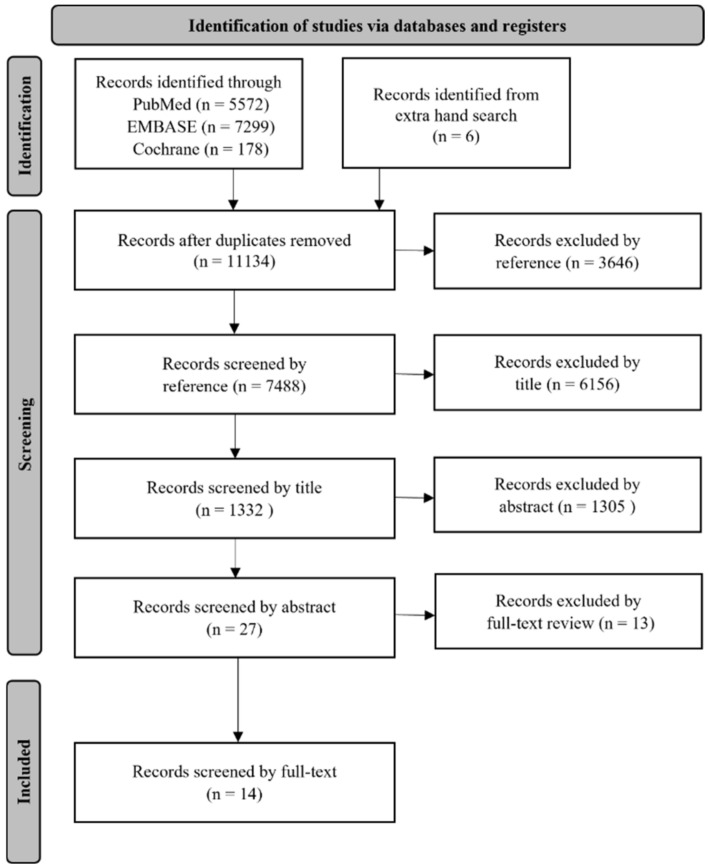
Flow diagram of the study selection process.

The detailed criteria for selecting and reviewing the articles are shown in Figure 1. The initial search from online databases yielded 13,049 records and six articles identified through a hand search. After removing duplicates, a total of 11,134 records remained. Following that, 3646 records were removed owing to an irrelevant reference type, which was reduced to 7488 records. Next, 6156 records were excluded by title, which was reduced to 1332 records. After 1305 records were removed by abstract, 27 records were selected for full-text review. In the process of full-text review, only 14 studies met the inclusion criteria and were included in the systematic review.

### 3.2. Yearly and Country-Wise Trend of Publication

**Figure 2:**
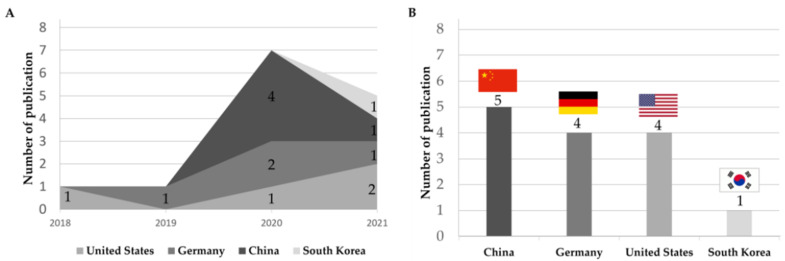
Publication trend of artificial intelligence-based microsatellite instability prediction models, (**A**) yearly and (**B**) country-wise.

The yearly and country-wise trends of publications are illustrated in Figure 2. The AI models for MSI prediction was first reported in 2018 and slightly increased so far. The included 14 studies were published from China (n = 5), followed by Germany (n = 4), the United States (n = 4), and South Korea (n = 1).

### 3.3. MSI Prediction Models by Cancer Types

**Figure 3:**
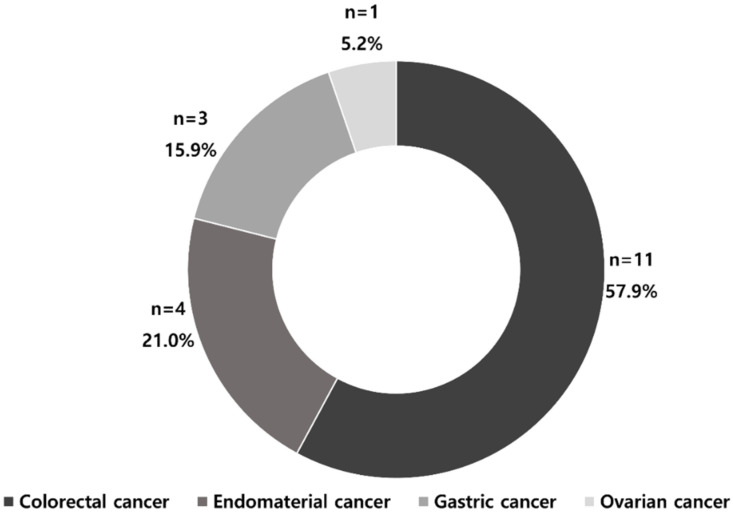
Artificial intelligence-based MSI prediction models according to target organs.

The number of publications on MSI models according to cancer types is shown in Figure 3. Most studies were from CRC (57.9%; n = 11), followed by endometrial (21.0%; n = 4), gastric (15.9%; n = 3), and ovarian cancers (5.2%; n = 1).

### 3.4. Prediction of MSI Status in CRC

The key characteristics of the AI models included in the CRC are summarized in Table 1. Most of the studies used the TCGA dataset for training and validation of their AI models. The study by Echle et al. used data from a large-scale international collaboration representing the European population for training, validation, and testing, which includes 6406 patients from Darmkrebs: Chancen der Verhütung durch Screening (DACHS), Quick and Simple and Reliable (QUASAR), and Netherlands Cohort Study (NLCS) datasets in addition to the TCGA dataset [30]. DACHS is a dataset of CRC patients with stage I-IV from the German Cancer Research Center. QUASAR is a clinical trial data of CRC patients, mainly with stage II tumors, from the United Kingdom. NLCS is a dataset from the Netherlands that includes patients of any tumor stage. The study by Lee et al. used an in-house dataset along with the TCGA dataset, and the study by Yamashita et al. used only an in-house dataset for training, validation, and testing of their AI models [48,49]. A study by Co et al. and Lee et al. used an Asian dataset for external validation, which is different from the population dataset used for training and testing their models [48,49].

**Figure 4:**
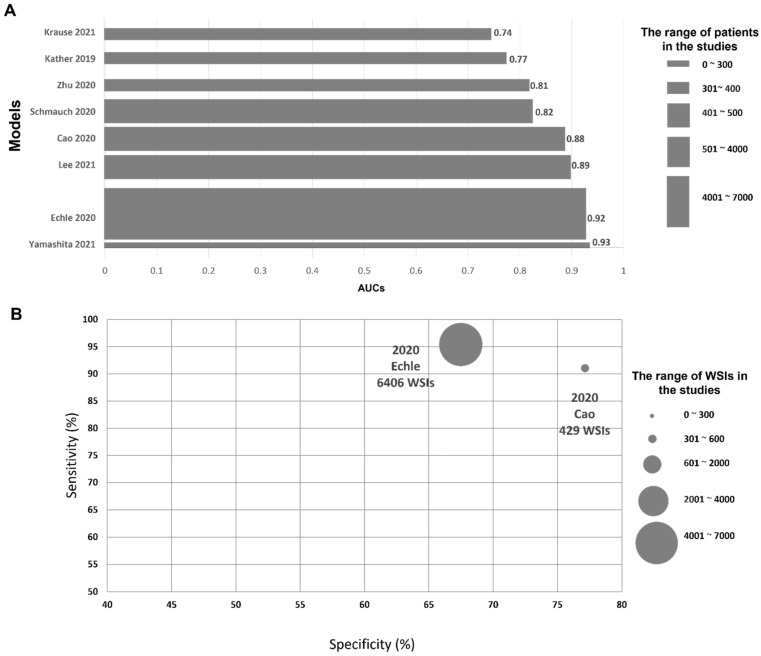
Comparison of the performance metric of microsatellite instability prediction models in colorectal cancers. (**A**). Area under the ROC curve. (**B**). Sensitivity and specificity.

The comparison of the AUC of their tests is shown in Figure 4. The performance metric AUC of AI models ranged 0.74–0.93. The highest AUC 0.93 was reported by Yamashita et al. with a small data set, but a study by Echle et al. with a large international dataset also showed good AUC 0.92. Kather et al. and Coa et al. trained and tested their models on frozen section slides (FSS) and compared their model performance with the results of a formalin-fixed paraffin-embedded (FFPE) slide dataset [29,50]. Their results showed that AUC is slightly higher in the model trained and tested on FSS in comparison to that trained and tested on FFPE.

A comparison of the sensitivity and specificity of the AI models of CRC is shown in Figure 4. Echle et al.’s study with a large-scale international dataset showed a good sensitivity of 95.0%, although its specificity was slightly low (67.0%) [30]. A study by Coa et al. showed good sensitivity and a specificity of 91.0% and 77.0%, respectively [50].

The type of AI models used for MSI prediction in each study is shown in the supplementary Table S1. We also compared the AUCs of AI models that used the same dataset and that is shown in Supplementary Figure S1A,B. Our data showed that the average performance of ResNet18 model in CRC was better in FSS (AUC 0.85) compared to FFPE (AUC 0.79). The next commonly used AI model for CRC was ShuffleNet, which was used by three studies. However, due to heterogeneity in their data, we were able to compare only two studies, which showed an average AUC of 0.83. The average AUCs of both ResNet18 and ShuffleNet classifiers were almost similar.

**Table 1:** Characteristics of the artificial intelligence models used for microsatellite instability prediction in colorectal cancers.

Author	Year	Country	AI Model	Training and Validation Data Set/WSIs/No. of Patients (n)	Pixel Levels	Additional Methodology for Validating MSI	Performance Metrics	External Validation Dataset/WSIs/No. of Patients (n)	External Validation Result	Ref.
Zhang	2018	USA	Inception-V3-	TCGA/NC/585	1000 × 1000	NC	ACC: 98.3%	NS	NS	[51]
Kather	2019	Germany	ResNet18	TCGA-FFPE/360/NC	NC	PCR	AUC: 0.77	DACHS-FFPE, n = 378	AUC: 0.84	[29]
				TCGA-FSS/387/NC	NC	PCR	AUC: 0.84	DACHS-FFPE, n = 378	AUC: 0.61	
Echle	2020	Germany	ShuffleNet	TCGA, DACHS, QUASAR, NLCS/6406/6406	512 × 512	PCR/IHC	AUC: 0.92 Specificity: 67.0% Sensitivity: 95.0%	YCR-BCIP-RESECT, n = 771	AUC: 0.95	[30]
								YCR-BCIP-BIOPSY, n = 1531	AUC: 0.78	
Cao	2020	China	ResNet18	TCGA-FSS/429/429	224 × 224	NGS/PCR	AUC: 0.88 Specificity: 77.0% Sensitivity: 91.0%	Asian-CRC-FFPE, n = 785	AUC: 0.64	[50]
Ke	2020	China	AlexNet	TCGA/747/NC	224 × 224	NC	MSI score: 0.90	NS	NS	[52]
Kather	2020	Germany	ShuffleNet	TCGA/NC/426,	512 × 512	PCR	NC	DACHS, n = 379	AUC: 0.89	[53]
Schmauch	2020	USA	ResNet50	TCGA/NC/465	224 × 224	PCR	AUC: 0.82	NS	NS	[54]
Zhu	2020	China	ResNet18	TCGA-FFPE: 360	NC	NC	AUC: 0.81	NS	NS	[55]
				TCGA-FSS: 385	NC	NC	AUC: 0.84			
Yamashita	2021	USA	MSINet	In-house sample/100/100	224 × 224	PCR	AUC: 0.93	TCGA/484/479	AUC: 0.77	[49]
Krause	2021	Germany	ShuffleNet	TCGA-FFPE, n = 398	512 × 512	PCR	AUC: 0.74	NS	NS	[56]
Lee	2021	South Korea	Inception-V3-	TCGA and SMH/1920/500	360 × 360	PCR/IHC	AUC: 0.89	NC	AUC: 0.97	[48]

Abbreviations: AI, artificial intelligence; DL, Deep learning; WSIs, whole slide images; TCGA, The Cancer Genome Atlas; DACHS, Darmkrebs: Chancen der Verhütung durch Screening; QUASAR, Quick and Simple and Reliable; NLCS, Netherlands Cohort Study; YRC-BCIP-RESECT, Yorkshire Cancer Research Bowel Cancer Improvement Programme-Surgical Resection; Yorkshire Cancer Research Bowel Cancer Improvement Programme-Endoscopic Biopsy Samples; Asian-CRC, Asian Colorectal Cancer Cohort; SMH, Seoul St. Mary’s Hospital; PCR, polymerase chain reaction; IHC, immunohistochemistry; NGS, next-generation sequencing; ACC, accuracy; AUC, area under the curve; FFPE, formalin-fixed paraffin-embedded; FSS, Frozen section slides; NC, not clear; NS, not specified.

### 3.5. Prediction of MSI Status in Endometrial, Gastric, and Ovarian Cancers

The key characteristics of the AI model studies on endometrial, gastric, and ovarian cancers are summarized in Table 2. In endometrial cancer, except for one study, all the other studies used only the TCGA dataset for the training, testing, and validation of their models. In addition to the TCGA dataset, Hong et al. used the Clinical Proteomic Tumor Analysis Consortium (CPTAC) dataset for training and testing [57]. This study also used the New York Hospital dataset for external validation. The performance metric AUC of the test ranged from 0.73–0.82. ResNet18 is also a commonly used AI model in endometrial cancer and comparison of their AUCs is shown in Supplementary Figure S1C.

All the included studies in gastric cancer used only the TCGA dataset for training, testing, and validation. The performance metric AUC of the test ranged from 0.76–0.81. Kather et al. reported that their model trained on mainly Western population data performed poorly in an external validation test with a dataset of the Japanese population [29]. ResNet18 is also a commonly used AI model in gastric cancer, and comparison of their AUCs is shown in the Supplementary Figure S1D.

Ovarian cancer included only one study, and this study used the TCGA dataset for training and testing for the AI model, with a performance metric of AUC 0.91 [58].

**Table 2:** Characteristics of the artificial intelligence models in endometrial, gastric, and ovarian cancers.

Organ /Cancers	Author	Year	Country	AI-Based Model	Data Set/WSIs/No. of Patients (n)	Pixel Level	Additional Methodology for Validating MSI	Performance Metrics	External Validation Dataset/WSIs/No. of Patients (n)	External Validation Result	Ref.
Endometrial cancer	Zhang	2018	USA	Inception-V3	TCGA-UCEC and CRC/1141/NC	1000 × 1000	NC	ACC: 84.2%	NS	NS	[51]
	Kather	2019	Germany	ResNet18	TCGA-FFPE/NC/492	NC	PCR	AUC: 0.75	NS	NS	[29]
	Wang	2020	China	ResNet18	TCGA/NC/516	512 × 512	NC	AUC: 0.73	NS	NS	[59]
	Hong	2021	USA	InceptionResNetVI	TCGA, CPTAC/496/456	299 × 299	PCR/NGS	AUC: 0.82	NYU-H/137/41	AUC: 0.66	[57]
Gastric cancer	Kather	2019	Germany	ResNet18	TCGA-FFPE/NC/315	NC	PCR	AUC: 0.81	KCCH-FFPE-Japan/NC/185	AUC: 0.69	[29]
	Zhu	2020	China	ResNet18	TCGA-FFPE/285/NC	NC	NC	AUC: 0.80	NS	NS	[55]
	Schmauch	2020	USA	ResNet50	TCGA/323/NC	224 × 224	PCR	AUC: 0.76	NS	NS	[54]
Ovarian cancer	Zeng	2021	China	Random forest	TCGA/NC/229	1000 × 1000	NC	AUC: 0.91	NS	NS	[58]

Abbreviations: AI, artificial intelligence; DL, Deep learning; WSIs, whole slide images; TCGA, The Cancer Genome Atlas; CPTAC, Clinical Proteomic Tumor Analysis Consortium; CRC, Colorectal Cancer; UCEC, Uterine Corpus Endometrial Carcinoma; NYU-H, New York University-Hospital; KCCH-Japan, Kanagawa Cancer Centre Hospital-Japan; ACC, accuracy; AUC, area under the ROC curve; NC, not clear; NS, not specified.

## 4. Discussion

In this study, we found that AI models for MSI prediction have been increasing recently, mainly focusing on CRC, endometrial, and gastric cancers, and the performance of these models is quite promising, but there were some limitations. More qualified data with external validation, including various ethnic groups, should be considered in future studies.

### 4.1. Present Status of AI Models

#### 4.1.1. Yearly, Country-Wise, and Organ-Wise Publication Trend

Yearly publication trends related to MSI prediction by AI are increasing, and most publications were from developed countries. A recent publication also suggested a similar trend on topics related to AI and oncology, which showed that the United States is the leading country, followed by South Korea, China, Italy, the UK, and Canada [60]. Publication trends related to overall AI research in medicine also showed exponential growth since 1998, and most papers were published between 2008 and 2018 [61]. In another report, the number of publications in overall AI and machine learning in oncology remained stable until 2014, but increased enormously from 2017 [60], which is consistent with our results.

**Figure 5:**
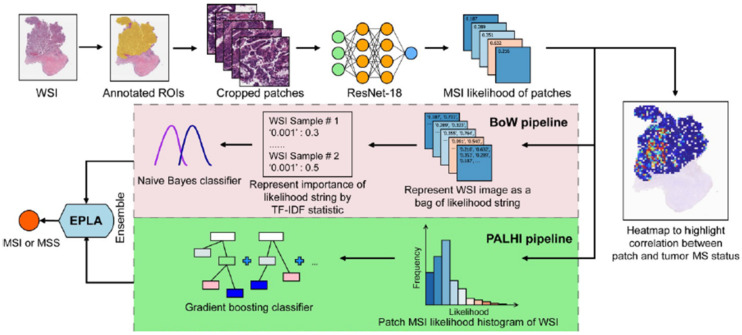
Example of an artificial intelligence model for colorectal cancer. Figure 1. Overview of the Ensemble Patch Likelihood Aggregation (EPLA) model. A whole slide image (WSI) of each patient was obtained and annotated to highlight the regions of carcinoma (ROIs). Next, patches were tiled from ROIs, and the MSI likelihood of each patch was predicted by ResNet-18, during which a heat map was shown to visualize the patch-level prediction. Then, patch likelihood histogram (PALHI) pipelines and bags of words (BoW) pipelines integrated the multiple patch-level MSI likelihoods into a WSI-level MSI prediction, respectively. Finally, ensemble learning combined the results of the two pipelines and made the final prediction of the MS status. Reprinted from Ref. [50].

Our data showed that the number of publications on MSI models is higher in CRC compared to endometrial, gastric and ovarian cancers. It may be because the CRC is the second most lethal cancer worldwide, and approximately 15% of CRC is caused by the MSI [6,7,8,9,62,63]. MSI-high tumors are widely considered to have a large neoantigen burden, making them especially responsive to immune checkpoint inhibitor therapy [64,65]. In recent years, MSI has gained much attention because of its involvement in predicting the response to immunotherapy for many types of tumors [66]. An example of the AI model for CRC is shown in Figure 5.

AI models using WSI showed great potential for prediction of MSI in CRCs, which can be used as a low-cost screening method for these patients. It also can be used as a prescreening tool to select MSI-H probability for patients before testing with the current costly available PCR/IHC methods. However, further validation of these models on a large dataset is necessary to improve their performance to an acceptable level of clinical usage. Most of the MSI models for CRC were developed on a dataset of surgical specimens. More models from endoscopic biopsy samples using more datasets from various ethnic populations should be developed in the future, which can reduce the possibility of missing MSI-H cases, particularly in advanced CRCs, where resection is not possible. Another limitation of these AI modes is that they cannot distinguish between hereditary and sporadic MSI cases. Therefore, to improve the performance of these models, training and validation with a large dataset is required in future research studies.

As immunotherapy and MSI testing gets more and more importance in other solid cancers such as gastric, endometrial, and ovarian cancers, we can see that the AI-based MSI prediction models have also been applied in these cancers recently. They showed promising results for a potential application, although the evidence is still insufficient. A large dataset with external validation should follow in the future.

#### 4.1.2. Performance of AI Models and Their Cost Effectiveness

The sensitivity and specificity of AI models were comparable to that of routinely used methods such as PCR and IHC. The study by Echle et al. and Coa et al. showed 91.0–95.0% of sensitivity and 67.0–77.0% of specificity [30,50]. In the literature, IHC sensitivity ranges from 85–100% and the specificity ranges from 85–92% [31,32]. MSI PCR showed 85–100% sensitivity and 85–92% specificity [31]. According to a recent study assessing the cost-effectiveness of these molecular tests and the AI models, the accuracy of MSI prediction models was similar to that of commonly used PCR and IHC methods [67]. NGS technology is useful for the testing of many gene mutations, such as for epithelial ovarian cancer patients with BRCA mutation or for HR deficiency that might benefit from a therapeutic option of platinum agents and PARP inhibitors, whereas immune checkpoint inhibitors are effective in tumors with the MSI-H [68].

**Figure 6:**
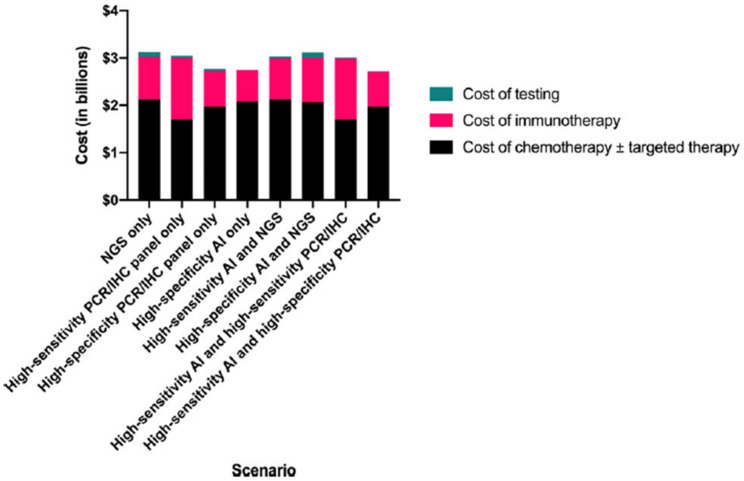
The cost effectiveness of MSI prediction models. Comparison of total testing and treatment-related costs by clinical scenario. AI, artificial intelligence; IHC, immunohistochemistry; NGS, next-generation sequencing; PCR, polymerase chain reaction. Reprinted from Ref. [67].

In this study, the authors predicted the net medical costs of six different clinical scenarios using the combination of different MSI testing methods including PCR, IHC, NGS and AI models and corresponding treatment in the United States. An overview of the cost effectiveness comparison of their study is shown in Figure 6. They reported that AI models with high PCR or IHC can save up to $400 million annually [67]. As the cancer burden is increasing, a precise diagnosis of MSI is essential to identify appropriate candidates for immunotherapy and to reduce the medical costs.

### 4.2. Limitation and Challenge of AI Models

#### 4.2.1. Data, Image Quality and CNN Architecture

To obtain the best results from any convolutional neural network (CNN) model, a large dataset from various ethnic groups is required for training, testing, and validation. Most studies in this review had a relatively small number of TCGA datasets for appropriate training and validation. Without a large-scale validation, the performance of these AI models cannot be generalized, and it is not feasible for routine diagnosis. One study could not perform further subgroup analysis due to limited clinical information of TCGA datasets [49]. Another study raised the potential limitation that the TCGA datasets may not represent the real situation [55]. Another group of researchers raised the potential limitation of technical artifacts such as blurred images in TCGA datasets [30]. Although the TCGA dataset includes patients from various institutes/hospitals, but all are the patients are from similar ethnic group, which is primarily from the North America. A few studies by Echle et al., Kather et al., Yamashita et al., and Lee et al. used European datasets (DACH) and local in-house datasets for training or external validation [29,30,48,49]. However, for high generalizability, the datasets from various ethnic groups should be explored further.

**Figure 7:**
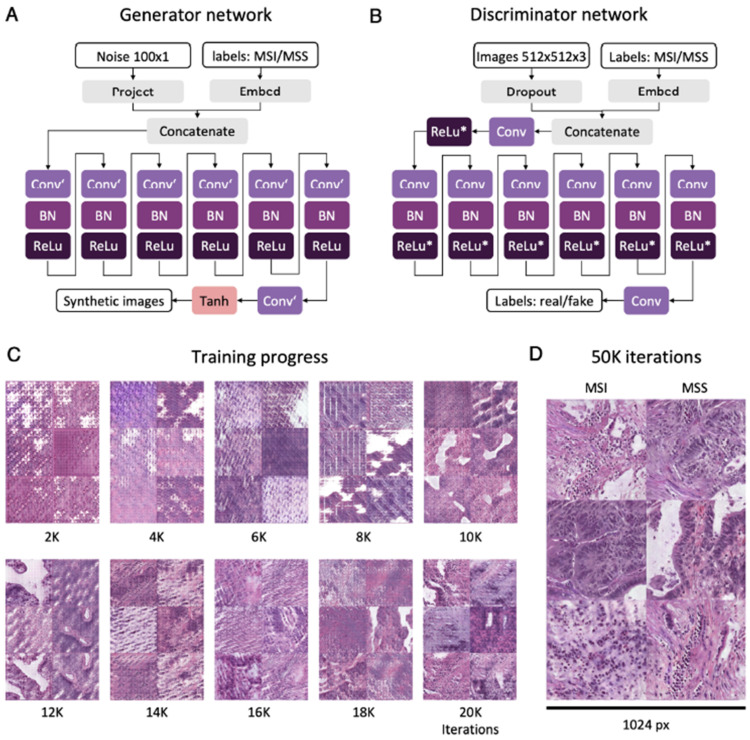
Overview of the conditional generative adversarial network study design. A conditional generative adversarial network (CGAN) for histology images with molecular labels. (**A**) Overview of the generator network for generation of synthetic histology image patches with 512 × 512 × 3 pixels. MSI, microsatellite instable; MSS, microsatellite stable; Conv’, transposed convolution 2D layer; BN, batch normalization layer; ReLu, rectified linear unit layer. (**B**) Overview of the discriminator network for classifying images as real or fake (synthetic). Conv, convolution 2D layer; ReLu*, leaky rectified linear unit layer. (**C**) Progress of synthetic images from 2000 (2K) to 20,000 (20K) epochs. (**D**) Final output of the generator network after 50,000 (50K) epochs. Reprinted from Ref. [56].

On a side note, one study reported poor performance with 40× magnification compared to 20× magnification which may be due to differences in the image color metrics [49]. Another study reported that the color normalization of images slightly improves performance of the AI model [30]. Cao et al. recommended to use the images over 20× magnification for a better performance [50]. Interestingly, Krause et al. in 2021 proposed a specialized method to train an AI model when only a limited number of datasets was available (Figure 7). They synthesized the 10,000 histological images with and without MSI using a generative adversarial network from 1457 CRC WSIs with MSI information [56]. They reported increased AUROC after adopting this method and an increase in the size of the training dataset, and this synthetic image approach can be used to for generating large datasets with rare molecular features.

The choice of CNN also affects the performance of the AI models; commonly used networks such as ResNet18, ShuffleNet, and Inception-V3 have been used in most of the studies. The ResNet model has many other variations as per the number of layers used, such as ResNet18, ResNet34, ResNet50, and many others. The ResNet18 model has 72-layer architecture with 18 deep layers, which may degrade the output result due to multiple deep layers in the network [69]. However, if the output result is degraded it can be fixed through back propagation. ShuffleNet has a simple design architecture, and it is also optimized for mobile devices [53]. Therefore, it can show good performance with a high accuracy at a low training time [53].

A study observed that lightweight neural network models performed on par with more complex models [53]. Performance comparison including three to six of these models is essential for enhancing the performance of the final model.

#### 4.2.2. External Validation and Multi-Institutional Study

In CRC cases, six out of 11 studies included an external validation. The performance metric AUC for external validation ranged from 0.61–0.97. In endometrial and gastric cancer cases, only one study for each group performed external validation. AI models that are trained and tested on a single dataset may overfit and perform well on internal datasets. However, these models show low performance when tested for external datasets. Therefore, external validation on different datasets is always necessary in order to have a well-trained AI model.

**Figure 8:**
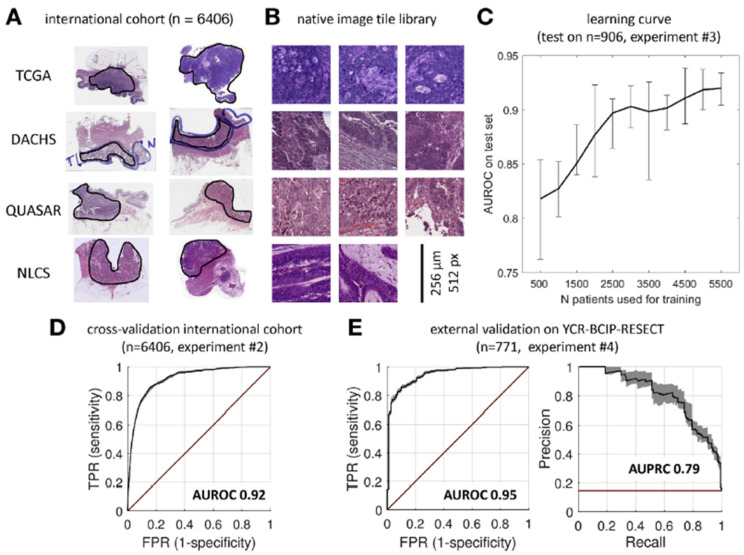
Overview of the multicentric study design. Deep learning workflow and learning curves. (**A**) Histologic routine images were collected from four large patient cohorts. All slides were manually quality checked to ensure the presence of tumor tissue (outlined in black). (**B**) Tumor regions were automatically tessellated, and a library of millions of nonnormalized (native) image tiles was created. (**C**) The deep learning system was trained on increasing numbers of patients and evaluated on a random subset (n = 906 patients). Performance initially increased by adding more patients to the training set but reached a plateau at approximately 5000 patients. (**D**) Cross validated experiment on the full international cohort (comprising TCGA, DACHS, QUASAR, and NLCS. The receiver operating characteristic (ROC) with true positive rate is shown against the false positive rate with the AUROC shown on top. (**E**) ROC curve (left) and precision-recall curve (right) of the same classifier applied to a large external data set. High test performance was maintained in this data set, and thus, the classifier generalized well beyond the training cohorts. The black line indicates average performance, the shaded area indicates bootstrapped confidence interval, and the red line indicates random model (no skill). FPR, false positive rate; TPR, true positive rate. Reprinted from Ref. [30].

Studies also suggested that a large sample size, multiple institutions data, and patients with different populations are needed to determine the generalization performance of their AI models. An overview of the multicentric study deign is shown in Figure 8. AI models trained mainly on data from Western populations performed poorly when validated on Asian populations [29]. Another study suggested that transfer learning for model fine-tuning in different ethnic populations may improve the generalizability of their AI models [50]. Previous researchers argued that datasets from multi-institutional and multinational models enhanced the generalizability of DL models [70,71].

#### 4.2.3. MSI Prediction on Biopsy Samples

Most studies only use WSIs of surgical specimens for the development of their AI models. However, MSI prediction on small colonoscopic biopsy samples is more practically useful in the clinical setting if it is feasible. A recent study observed relatively low performance on biopsy samples with their surgical specimen trained AI model [30]. Thus, further research on small biopsy samples is required to increase the performance.

#### 4.2.4. Establishment of Central Facility

AI technology in medical applications is still growing recent study showed increasing trend of patent related to AI and pathological images [72]. The lack of installed slide scanners in hospitals can hinder the implementation of DL models. The WSIs are large files which cannot be stored in a routine hospital setting. The whole slide scanners and the viewing and archiving system along with an appropriate server is expensive equipment that cannot be easily established. The establishment of central slide scanner facilities with a server with a larger data storage capacity can overcome this challenge [45,73].

### 4.3. Future Direction

Originally, AI applications in the pathology field focused on mimicking or replacing human pathologists’ tasks, such as segmentation, classification, and grading. The main goal of these studies was to reduce intra- or inter-observer variability in pathologic interpretation to support or augment human ability.

AI models trained with small datasets may overfit the target sample and may adversely affect the performance. For the accuracy of AI models, factors such as class imbalance and selection bias of the dataset must be considered during the development of the models. Since labels of the datasets are important for the training of AI models, biased and low-quality labeled datasets will decrease the performance of AI models. Therefore, collaborative research work between pathologists and AI researchers is needed. Furthermore, most of the studies used the TCGA dataset, which is a collection of representative cases, and may not efficiently represent the general population. Therefore, their performance cannot be generalized to the population as it may not contain many rare morphologic types of samples that exist in the general population. For the future, we suggest collecting a larger dataset of various ethnic populations, reviewed by experienced pathologists to minimize the selection bias and enhance the generalizability of AI models. Furthermore, external validation should be performed with the representative data of various ethnic populations. Randomized controlled trials are a useful tool to assess the risk and benefit in medical research studies. There is a need for randomized clinical studies or prospective clinical trials for AI models before using these models for routine clinical practice. Most of the AI models were developed using surgical sample datasets. Despite immunotherapy being the best treatment choice for CRC patients with stage IV tumors, the endoscopic biopsy sample is the only available tissue from these patients due to the inability of surgical resection. Future studies are needed to accurately estimate MSI based on biopsy samples, which will aid in the selection of immunotherapy for patients with advanced CRC cancer. Currently available AI models can not specifically differentiate between Lynch syndrome and MSI-H in sporadic cancer patients. The Development of an AI model for detecting Lynch syndrome may help in selecting better therapeutic options for these patients. It is difficult to understand how the AI models arrive at a conclusion. This is because AI algorithms process data in a “black box”. Therefore, the AI models should be validated against the currently available quality standards to ensure their efficiency.

However, scientists are increasingly focusing on the “superpower” from AI models that can surpass human abilities, such as mutation, prognosis, and treatment response predictions in cancer patients. Our research group has already developed an AI model for MSI prediction in CRC, and the results is quite promising [48]. These findings motivated us to initiate a multi-institutional research project for the MSI prediction from CRC WSIs. Our first aim is to collect a large image dataset of CRC patients and verify the quality of the image by experienced pathologists. Second, we will develop an AI model using this large image dataset and test the generalized performance of AI models so that it may be feasible to use it in routine practice. At present, we are in the process of scanning the H&E slides of CRC patients in collaboration with 14 hospitals/institutions around the country.

## 5. Conclusions

This study showed that in the future, AI models can be an alternative and effective method for the prediction of MSI-H from WSIs. Overall, AI models showed promising results and have the potential to predict MSI-H in a cost-effective manner. However, the lack of a large dataset, multiethnic population sample, and lack of external validation were major limitations of the previous studies. Currently, the AI models are not approved for clinical use to replace routine molecular tests. As the cancer burden is increasing, there is need for the precise diagnostic method for predicting MSI-H and identify appropriate candidates for immunotherapy and to reduce the medical costs. AI models also can be used as a prescreening tool to select MSI-H probability for patients before testing with the current costly available PCR/IHC methods. Future studies are needed to accurately estimate MSI based on biopsy samples, which will aid in the selection of immunotherapy for patients advance stages of CRC. Moreover, currently available AI models can not specifically differentiate between Lynch syndrome and MSI-H in sporadic cancer patients. The development of an AI model for detecting Lynch syndrome may help in selecting better therapeutic options for these patients. As a result, to ensure efficiency, AI models should be tested against currently existing quality standards before being used in clinical practice. Well-designed AI models in the future can improve their performance without compromising diagnostic accuracy. Training and validation with a larger dataset and external validation on new datasets may improve the performance of AI models to an acceptable level.

## Data Availability

The data presented in this study are available upon request from the corresponding author (https://www.researchgate.net/profile/Yosep-Chong (accessed on 17 April 2022)). The data are not publicly available due to institutional policies.

## Supplementary Materials

The following supporting information can be downloaded at: https://www.mdpi.com/article/10.3390/cancers14112590/s1, Figure S1: Comparison of AUCs of AI models. (A) Comparison of ResNet18 in colorectal cancer. (B) comparison of ShuffleNet in colorectal cancer. (C) Comparison of ResNet18 in endometrial cancer. (D) Comparison of ResNet18 in gastric cancer; Table S1: Artificial intelligence models used for microsatellite.
