# Supplementary material for: Recent Applications of Artificial Intelligence from Histopathologic Image-Based Prediction of Microsatellite Instability in Solid Cancers: A Systematic Review
Source: Cancers (Basel). 2022 May 24;14(11):2590. doi: 10.3390/cancers14112590 (PMC9179592; doi:10.3390/cancers14112590)
Supplement: Supplementary file 1 [file cancers-14-02590-s001.zip › cancers-1709742-supplementary.pdf]

cancers

MDPI

Supplementary Materials

Recent Applications of Artificial Intelligence from Histopathologic Image-Based Prediction of Microsatellite Instability in Solid Cancers: A Systematic Review

Mohammad Rizwan Alam, Jamshid Abdul-Ghafar, Kwangil Yim, Nishant Thakur, Sung Hak Lee, Hyun-Jong Jang, Chan Kwon Jung and Yosep Chong

Table S1. Artificial intelligence models used for microsatellite instability prediction in each study.

Cancer type	AI models	Authors	Ref.
Colorectal cancer	ResNet18	Kather et al.	[29]
		Cao et al.	[50]
		Zhu et al.	[55]
	ShuffleNet	Echle et al.	[30]
		Kather et al.	[53]
		Krause et al.	[56]
	Inception-V3	Zhang et al.	[51]
		Lee et al.	[48]
	ResNet50	Schmauch et al.	[54]
	AlexNet	Ke et al.	[52]
	MSINet	Yamashita et al.	[49]
Endometrial cancer	ResNet18	Kather et al.	[29]
		Wang et al.	[59]
	Inception-V3	Zhang et al.	[51]
	InceptionResNetVI	Hong et al.	[57]
Gastric cancer	ResNet18	Kather et al.	[29]
		Zhu et al.	[55]
	ResNet50	Schmauch et al.	[54]
Ovarian cancer	Random forest	Zeng et al.	[58]

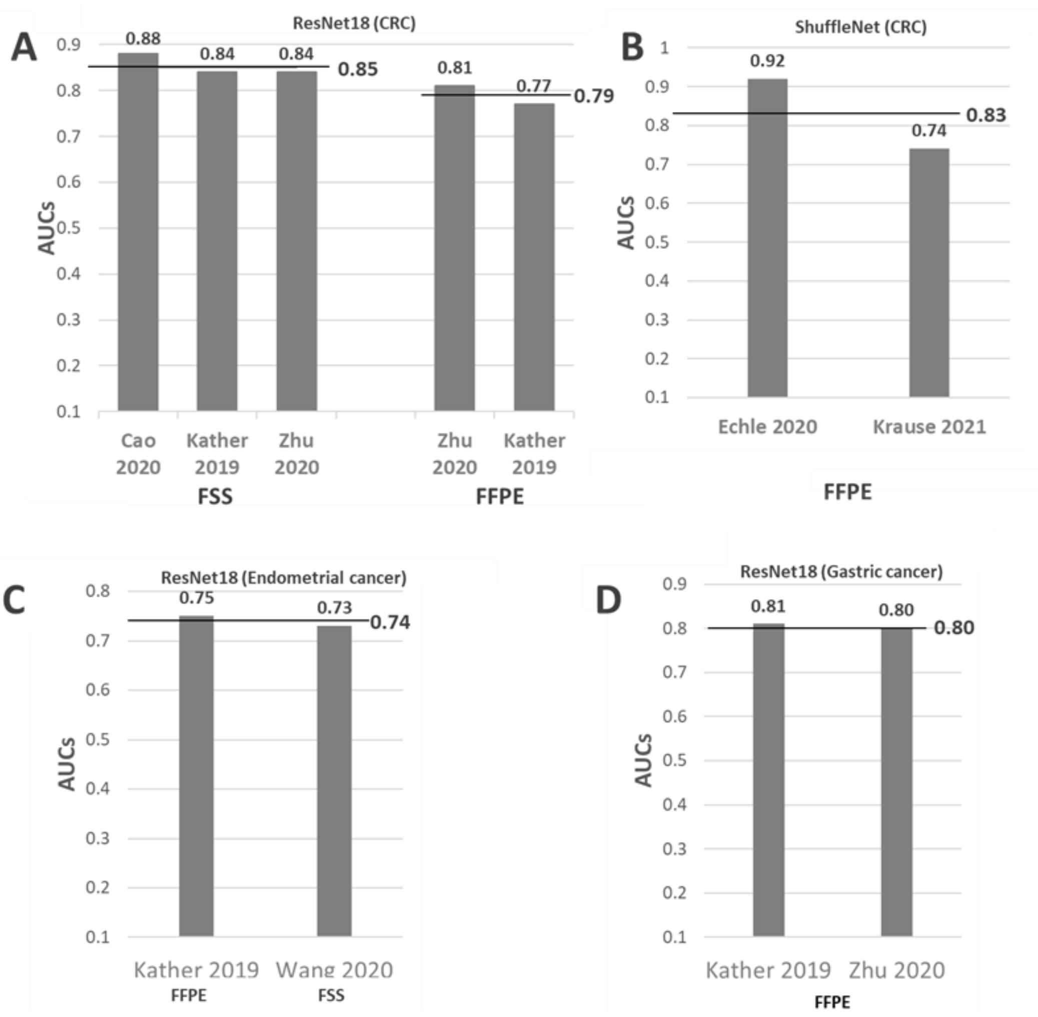

Figure S1. Comparison of AUCs of AI models. (A) Comparison of ResNet18 in colorectal cancer. (B) comparison of ShuffleNet in colorectal cancer. (C) Comparison of ResNet18 in endometrial cancer. (D) Comparison of ResNet18 in gastric cancer.
